# Post-COVID-19 arthritis: a case report and literature review

**DOI:** 10.1007/s10067-020-05550-1

**Published:** 2021-02-15

**Authors:** M. Gasparotto, V. Framba, C. Piovella, A. Doria, Luca Iaccarino

**Affiliations:** 1 Rheumatology Unit, Department of Medicine-DIMED, University of Padua, Padua, Italy; 2 Internal Medicine Unit and Regional Liver Disease Reference Centre, Department of Medicine – DIMED, University of Padua, Padua, Italy; 3 Division of Rheumatology, University of Padova, Via Giustiniani, 2, 35128 Padova, Italy

**Keywords:** Arthritis, COVID-19, Molecular mimicry, Reactive arthritis, SARS-CoV2

## Abstract

Severe acute respiratory syndrome coronavirus 2 (SARS-CoV2) is the novel pathogen responsible for the coronavirus disease 19 (COVID-19) outbreak. Researchers and clinicians are exploring the pathogenetic mechanisms of the viral-induced damage and growing interest is focusing on the short-term and long-term immune-mediated consequences triggered by the infection. We will focus on post-SARS-CoV2 infection arthritis which may arise as a new pathological condition associated with COVID-19. In this article, we describe a case of acute oligoarthritis occurring 13 days after a SARS-CoV2 severe pneumonia in a middle-aged Caucasian man and we go over a brief review of the current available literature. We hypothesize that molecular mimicry might be the basic immunological mechanism responsible for the onset of COVID-19-related arthritis based on the current knowledge of SARS-CoV2 and on the known pathogenetic mechanism of viral-induced arthritis.

## Introduction

In the midst of COVID-19 outbreak, researchers from all over the world are studying the pathogenetic mechanisms of SARS-CoV2 respiratory infection but growing interest is also focusing on the immune-mediated consequences that could be secondarily triggered by the virus. The most severe cases are characterized by a marked pro-coagulant state [1, 2] and by an inflammatory cytokine storm similar to that found in macrophage activation syndrome [3–5]. A dysregulated hyperimmune response definitely contributes to the severity of damage and may elicit autoimmune processes in predisposed individuals. Viral infections are supposed to be involved in the pathogenesis of many rheumatological conditions and several cases of autoimmune-induced diseases after SARS-CoV2 infection are reported in the literature [6–9].

## Case report description

**Fig. 1:**
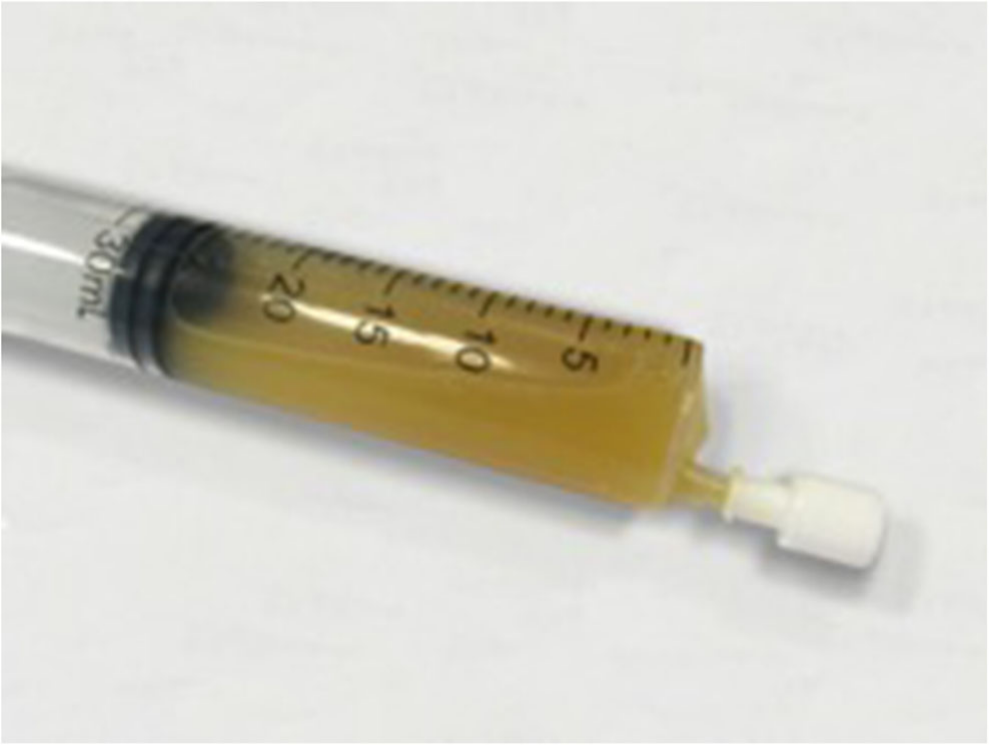
Patient’s synovial fluid

**Fig. 2:**
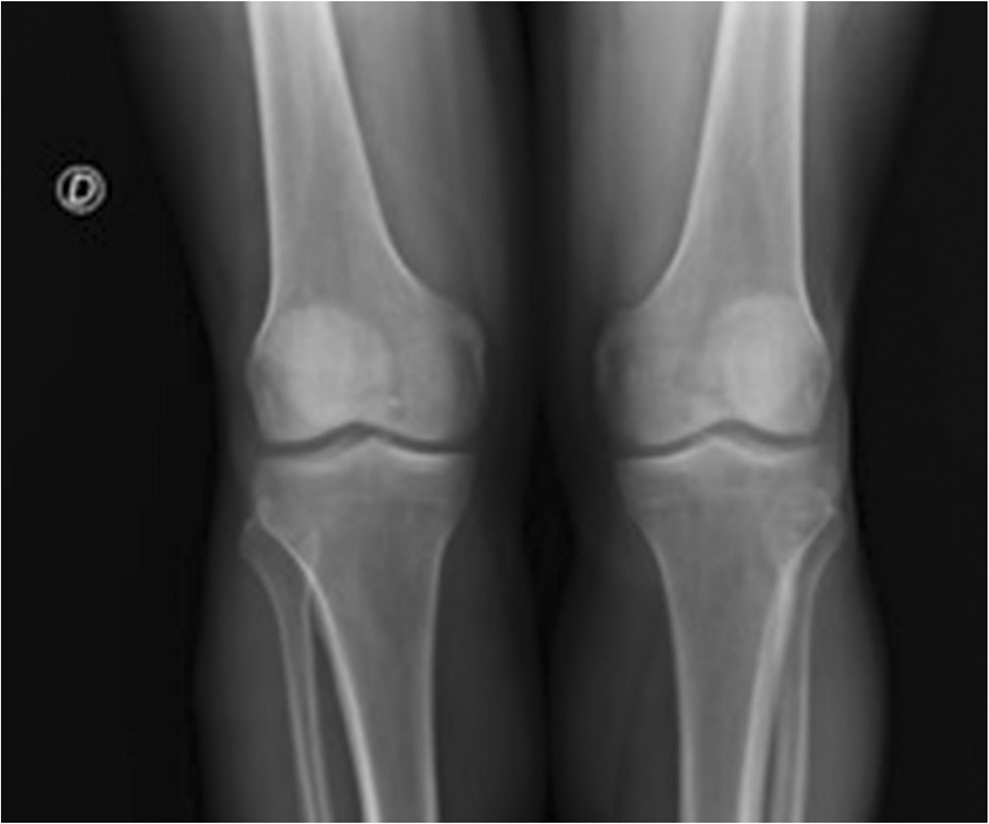
Knees X-ray

We describe a case of acute arthritis after a SARS-CoV2 infection in a 60-year-old Caucasian man without relevant comorbidities (Table 1). In April 2020, he was hospitalized for hyperpyrexia, headache, asthenia, and a worsening dyspnea. At the emergency room (ER), the thoracic ultrasound and chest X-ray revealed an interstitial pneumonia; a nasopharyngeal swab was positive for SARS-CoV2 and his blood test revealed a marked inflammatory state characterized by CRP (C-reactive protein) 240 mg/L, fibrinogen 9.83 g/L, interleukin-6 162 ng/L, ferritin 944 μg/L, and D-dimer 993 μg/L. He was admitted to the Internal Medicine department and treated with azithromycin, ceftriaxone, hydroxychloroquine (HCQ) (400 mg/die), anticoagulation for thromboembolism prophylaxis, and low-flow oxygen. For progressive respiratory failure, he was referred to the intensive care unit where he underwent nasotracheal intubation and received broad-spectrum antibiotics (meropenem, linezolid), antimycotic prophylaxis, continuous diuretic infusion, noradrenalin for hemodynamic support, and therapeutic dose of anticoagulants for elevation in D-dimer values. Due to a progressive improvement of respiratory gas exchange and chest X-ray, he was extubated after 10 days. He was discharged in good general conditions and low-grade inflammation on blood tests after overall 19 days of hospitalization. The weekly surveillance nasopharyngeal swabs for SARS-CoV2 persisted negative. Nevertheless, 13 days after discharge, he complained tenderness of the right ankle, knee, and hip in association with low-grade fever. He presented to the ER with oligoarthritis of the right lower limb and high CRP level (237 mg/L) on blood tests. Physical and ultrasound examination confirmed slight right ankle inflammation and clear right knee arthritis. Arthrocentesis led to the evacuation of 20 cc of a cloudy, yellow, and highly inflammatory synovial fluid (SF) (Fig. 1) whose analysis revealed 20.000/mm^3^ white blood cells of which 90% polymorphonucleates and 10% monocytes; no crystals were detected. Synovial RT-PCR (real-time polymerase chain reaction) for SARS-CoV2, as well as SF culture for bacterial agents, was negative (Table 2). He denied any infectious symptom, recent history of physical trauma, dyspnea, previous episode of arthritis, dactylitis, conjunctivitis or uveitis nor inflammatory diarrhea, and personal or familial history of psoriasis. To proceed with further investigations, the patient was hospitalized again. A new nasopharyngeal swab and a negative research of SARS-CoV2 nucleic acid on sputum excluded a recurrence of the systemic infection while serology showed indisputable seroconversion (a-SARS-CoV2 IgG 37.120 KUA/L and a-SARS-CoV2 IgM 9.163 KUA/L). Urine and blood cultures were negative and procalcitonin within the normal range; urethral swab and stool culture did not show evidence of bacterial infection. The in-depth examination for systemic rheumatic causes of arthritis were negative including antinuclear antibodies (ANA), extractable antinuclear antibodies, rheumatoid factor (RF), anti-citrullinated peptide, and HLA-B27 typing (Table 3). The knees, ankles, and hip X-ray did not show erosions or intra-articular calcifications (Fig. 2). Even though the patient’s SF was markedly inflammatory, which is an infrequent finding in infectious-related arthritis, the temporal relation with SARS-CoV2 infection made the hypothesis of post-viral acute arthritis the most probable. A nonsteroidal anti-inflammatory (NSAID) therapy with ibuprofen 600-mg; bid was started with clinical benefit and decrease of CRP. The patient was discharged after 9 days and continued the NSAIDs for other 3 weeks. Up to 6 months after therapy discontinuation, he presented no signs of arthritis recurrence.

**Table 1:** Laboratory findings during clinical course in our patient

	Day of discharge for COVID-19 hospitalization	Day of hospitalization for oligoarthritis arthritis	Day of discharge
WBCs (4400–11.000/mmc)	6490	9020	5510
Neutrophils (1.800–7.800/mmc)	2750	5750	2990
Lymphocytes (1.100–4800/mmc)	2080	990	1760
Monocytes (200–960/mmc)	910	630	560
Eosinophils (0–500/mmc)	690	220	190
Basophils (0–200 /mmc)	6	10	4
Hb (14–17.5 g/dL)	11.8	11.8	11.7
PLTs (150.000–410.000/mmc)	380.000	330.000	388.000
ESR (2–37 mm/h)	-	111	72
CRP (0–6 mg/L)	12	237	14
D-dimer (0–300 μg/L)	1490	839	-
Fibrinogen (1.5–4.5 g/L)	7.66	10.61	-
LAD (135–225 U/L)	260	213	167
Ferritin (20–250 μg/L)	700	446	248
Procalcitonin (0–0.5 μg/L)	< 0.04	< 0.04	-
SARS-CoV2 swab	Negative	Negative	Negative

*WBC* white blood cell, *Hb* hemoglobin, *PLT* platelet, *ESR* erythrocyte sedimentation rate, *CRP* C-reactive protein, *LAD* lactate dehydrogenase, *SARS-CoV2* severe acute respiratory syndrome coronavirus 2

**Table 2:** Synovial fluid analysis

Aspect	Yellow turbid
Differential count of WBC	WBC 20.000/mmc, PMN 90%, M 10%
Polarized light microscopy	No crystals
SARS-Cov2 RT-PCR	Negative
Culture	Negative

*WBC* white blood cell, *PMN* polymorphonucleate, *M* monocyte, *RT-PCR* real-time polymerase chain reaction

**Table 3:** Case reports of arthritis reactive to SARS-CoV2 infection, clinical characteristics and diagnostic workup

	***Yokogawa et al. [10]***	***Liew et al. [11]***	***Ono et al. [12]***	***Saricaoglu et al. [13]***	***Danssaert et al. [14]***	***Parisi et al. [15]***	***Present case***
**Age and sex**	57-year-old man	47-year-old man	Male in his 50s	73-year-old man	37-year-old female	58-year-old female	60-year-old man
**Time to arthritis**	15 days after COVID19 diagnosis	At diagnosis of COVID19	21 days after COVID19 diagnosis	15 days after COVID19 diagnosis	12 days after COVID19 diagnosis	25 days after prodrome infective symptoms	32 days after COVID19 diagnosis
**Affected joints**	Right knee	Right knee	Left and right ankle	Left I MTP, PIP, DIP and right II PIP and DIP	Tendonitis of the II, III and IV extensor of the right hand	Ankle	Right knee and ankle
**Therapy**	No therapy (self-recovery)	Oral NSAID and intra-articular corticosteroid	Oral NSAID and intra-articular corticosteroid	Oral NSAID	Topical NSAID, oral opioid, gabapentin	Oral NSAID	Oral NSAID
**SF polarised microscopic examination**	No crystals	No crystals	No crystals	-	-	-	No crystals
**SF culture**	-	(negative Gram stain)	Negative	-	-	-	Negative
**SF PCR for SARS-CoV2**	Negative	Negative	-	-	-	-	Negative
**M. pneumoniae and C. pneumoniae serology**	-	-	Negative	-	-	-	Negative
**Gonococco PCR/serology**	Negative	Negative	Negative	-	-	-	-
**C. thracomatis PCR/serology**	-	Negative	Negative	-	-	-	-
**M. urealyticum PCR/serology**	-	-	-	-	-	-	-
**Urethral swab**	-	-	-	-	-	-	Negative
**Uric acid**	-	-	Within the normal range	Within the normal range	Within the normal range	-	Within the normal range
**RF**	-	-	Negative	Negative	Negative	Negative	Negative
**ACPA**	-	-	Negative	Negative	-	Negative	Negative
**ANA**	-	-	Negative	-	Negative	Negative	Negative
**Anti-ENA**	-	-	-	-	-	Negative	Negative
**HLA-B27**	-	-	Negative	-	-	Negative	Negative
**HBV and HCV antibodies**	-	-	Negative	-	-	-	Negative
**CMV-DNA and EBV-DNA**	-	-	-	-	-	-	< 1000 copies/mL
**Urine culture**	-	-	-	-	-	-	Negative
**Blood culture**	-	-	-	-	-	-	Negative
**Stool culture**	-	-	-	-	-	-	Negative

*COVID19, Coronavirus disease 19; MTP, metatarsophalangeal, PIP, proximal interphalangeal; DIP, distal interphalangeal; NSAID, non-steroidal anti-inflammatory drug; SF, synovial fluid; PCR, polymerase chain reaction; SARS-CoV2, severe acute respiratory syndrome coronavirus 2; RE, rheumatoid factor; ACPA, anti-citrullinated peptide antibodies; ANA, anti-nuclear antibodies; ENA, Extractable nuclear antibodies; HLA, human leukocyte antigen, HBV, hepatitis B virus; HCV, hepatitis C virus*

## Discussion and review of the literature

The pathogenesis of viral associated arthritis is only partially understood but one of the mechanisms supposed to mediate the activation of the inflammatory process is molecular mimicry [16], well known to be responsible for eliciting autoimmune responses in predisposed individuals [17, 18]. Examples of molecular mimicry concerning SARS-CoV2 are reported [18] and this mechanism is hypothetically involved in the pathogenesis of both the acute systemic infection and the post-infective viral-related immunological consequences [19, 20]. Previous and actual studies demonstrate that coronaviruses share molecular epitopes with human proteins (e.g., spike glycoprotein S) that play a key role to host cell invasion and escape immune response attacks, giving to the infectious agent an immune-evasive capacity [21, 22]. SASR-CoV2 shares three sequences of six amino acids with as many brainstem human proteins and the cross-reaction between human and viral epitopes may lead to brainstem damage and respiratory failure [23]. Other suggestive examples of molecular mimicry-driven diseases after COVID-19 come from recent publications reporting cases of Guillain-Barre and Miller-Fisher syndrome [24, 25]. Mimicking epitopes may also be present in synovial membrane and cause, with similar mechanism, an acute local inflammation.

To study in depth the relationship between SARS-CoV2 infection and post-COVID-19 arthritis, we performed a comprehensive search on PubMed of all the reported cases of acute arthritis in patients with SARS-CoV2 infection from January 2020 to October 2020 combining the following keywords: acute arthritis, reactive arthritis, viral arthritis, COVID-19, coronavirus, and SARS-CoV2. We considered only English-written case reports of adult patients. Thirteen articles met our searching inclusion criteria; four were excluded because they are not pertinent with the purpose of our review, one because it was a correspondence letter to an already considered article with no case report included in the text, and two because of describing cases of drug-induced gouty arthritis or patients with previous history of gout and therefore not strictly categorizable in among the group of post-COVID-19 arthritis. The six articles which satisfied inclusion and exclusion criteria refer to 6 case reports [10–15] which are included in Table 3.

The low prevalence of this clinical condition we found in patients with COVID-19 could be due to the use of HCQ and corticosteroids for the treatment of the viral infection which may prevent or weaken the inflammatory joint manifestations. Despite HCQ has not demonstrated to be effective in the treatment of COVID-19 [26], it has proven efficacy in the management of systemic rheumatological diseases, especially with inflammatory joint involvement. HCQ is the anchor drug in systemic lupus erythematosus, acting as immunomodulator, and prevents or mitigates lupus clinical manifestation in autoantibody positive asymptomatic subjects [27]; it is also part of the treatment for the milder forms of rheumatoid arthritis and Sjogren syndrome with frequent episodes of joint pain [28].

Analyzing more specifically the six cases of suspected COVID-19-related arthritis, it is apparent how the SF analysis was not performed in three cases [13–15]; therefore a microcrystalline etiology cannot be certainly ruled out. In the remaining three cases [10–12], monosodium urate and calcium pyrophosphate crystals were not detected at polarized light microscope examination, thus configuring an essential step forward in the exclusion diagnostic process. No other chemical-physical characteristics, including the number of white blood cells and their differential count, have been reported. As shown in Table 3, the lag time between SARS-CoV2 infection and onset of arthritis is variable but joint symptoms generally present days after the acute viral infection and usually during the healing period. Clinical and epidemiological data show a prominent involvement of lower limb joints with mono- or oligoarticular symptoms and a predilection for male sex. The clinical presentation that emerges from these case reports may deviate from the classic picture of a viral-related arthritis where joint involvement usually occurs during the viremia period and presents with a polyarticular pattern sometimes resembling rheumatoid arthritis [29].

Viral-related arthritis remains, in the most cases, a diagnosis of exclusion and this underlies the importance to exhaustively perform all the tests to rule out other possible diagnosis. Unfortunately, the diagnostic workup carried out in the reported cases is often partial and incomplete. In particular, a broad microbiological investigation comprehensive of blood, urine and stool cultures, urethral swab, and serological tests for bacteria responsible for reactive arthritis is in some cases lacking. By contrast, in our case report, the chemical-physical characteristics and microbiologic cultures of SF excluded septic and microcrystalline arthritis; broad-spectrum microbiological and serological tests for the common agent of bacterial reactive arthritis did not show any evidence of active or recent infections; the autoantibody immune profile resulted negative too. Finally, RT-PCR for the detection of SARS-CoV2 nucleic acids did not show the presence of the virus in the SF and this validates the hypothesis of an immune-mediated process.

Interestingly, all these suspected post-COVID-19 arthritis cases share a complete and prompt response to NSAID and/or glucocorticoids that, together with the onset timing and joint localization of arthritis, play in favor of a strict relation with SARS-CoV2 infection.

If we suppose a molecular mimicry-based pathogenesis, where the antibody response to the virus is crucial to induce joint inflammation, the rapid lowering of post-infection immunity along weeks could in turn have contributed to the fading of arthritic manifestations. Finally, this pathogenetic hypothesis, based on immune system hyperactivation, could also explain why arthritis has been reported only in patients with a severe infection; in milder forms of COVID-19, joint involvement may have a subclinical course and therefore less frequently come to medical attention.

## Conclusions

Before COVID-19 outbreak, no cases of coronavirus-related arthritis have been reported in literature but SARS-CoV2 represents a new devastating entity still under study on worldwide scale. Many steps forward in the comprehension of the viral pathogenicity have been made since the start of the pandemic, but much of the infection-related consequences remain to be discovered. A growing number of cases of COVID-19-related arthritis are being reported in literature, configuring this condition worthy of further study. Complete clinical and laboratory data, SF analysis, and a strict follow-up of the patient are of paramount importance to perform a careful differential diagnosis and to better define the characteristic of inflammatory joint involvement related to SARS-CoV2 infection.

## Data Availability

All data relevant to the clinical case are included in the article.

## References

[1] Spiezia L, Boscolo A, Poletto F, Cerruti L, Tiberio I, Campello E, Navalesi P, Simioni P (2020). COVID-19-related severe hypercoagulability in patients admitted to intensive care unit for acute respiratory failure. Thromb Haemost.

[2] Wichmann D, Sperhake JP, Lütgehetmann M, Steurer S, Edler C, Heinemann A, Heinrich F, Mushumba H, Kniep I, Schröder AS, Burdelski C, de Heer G, Nierhaus A, Frings D, Pfefferle S, Becker H, Bredereke-Wiedling H, de Weerth A, Paschen HR, Sheikhzadeh-Eggers S, Stang A, Schmiedel S, Bokemeyer C, Addo MM, Aepfelbacher M, Püschel K, Kluge S (2020). Autopsy findings and venous thromboembolism in patients with COVID-19: a prospective cohort study. Ann Intern Med.

[3] McGonagle D, Sharif K, O’Regan A (2020). The role of cytokines including interleukin-6 in COVID-19 induced pneumonia and macrophage activation syndrome-like disease. Autoimmun Rev.

[4] Bindoli S, Felicetti M, Sfriso P, Doria A (2020). The amount of cytokine-release defines different shades of Sars-Cov2 infection. Exp Biol Med.

[5] Henderson LA, Canna SW, Schulert GS, Volpi S, Lee PY, Kernan KF, Caricchio R, Mahmud S, Hazen MM, Halyabar O, Hoyt KJ, Han J, Grom AA, Gattorno M, Ravelli A, Benedetti F, Behrens EM, Cron RQ, Nigrovic PA (2020). On the alert for cytokine storm: immunopathology in COVID-19. Arthritis Rheum.

[6] Jones VG, Mills M, Suarez D, Hogan CA, Yeh D, Segal JB, Nguyen EL, Barsh GR, Maskatia S, Mathew R (2020). COVID-19 and Kawasaki disease: novel virus and novel case. Hosp Pediatr.

[7] Viner RM, Whittaker E (2020). Kawasaki-like disease: emerging complication during the COVID-19 pandemic. Lancet.

[8] Harzallah I, Debliquis A, Drénou B (2020). Lupus anticoagulant is frequent in patients with Covid-19. J Thromb Haemost.

[9] Andina D, Noguera-Morel L, Bascuas-Arribas M, Gaitero-Tristán J, Alonso-Cadenas JA, Escalada-Pellitero S, Hernández-Martín Á, Torre-Espi M, Colmenero I, Torrelo A (2020). Chilblains in children in the setting of COVID-19 pandemic. Pediatr Dermatol.

[10] Yokogawa N, Minematsu N, Katano H, Suzuki T (2020) Case of acute arthritis following SARS-CoV2 infection. Ann Rheum Dis 0:1. 10.1136/annrheumdis-2020-218281 10.1136/annrheumdis-2020-218281 32591356

[11] Liew IY, Mak TM, Cui L, Vasoo S, Lim XR (2020). A case of reactive arthritis secondary to coronavirus disease 2019 infection. J Clin Rheumatol.

[12] Ono K, Kishimoto M, Shimasaki T, Uchida H, Kurai D, Deshpande GA, Komagata Y, Kaname S (2020). Reactive arthritis after COVID-19 infection. RMD Open.

[13] Saricaoglu EM, Hasanoglu I, Guner R (2020) The first reactive arthritis case associated with COVID-19 [Online ahead of print]. J Med Virol. 10.1002/jmv.26296 10.1002/jmv.26296 PMC7405389 32652541

[14] Danssaert Z, Raum G, Hemtasilpa S (2020). Reactive arthritis in a 37-year-old female with SARS-CoV2 infection. Cureus.

[15] Parisi S, Borrelli R, Bianchi S, Fusaro E (2020). Viral arthritis and COVID-19. Lancet Rheumatol.

[16] Fujinami RS, von Herrath MG, Christen U, Whitton JL (2006). Molecular mimicry, bystander activation, or viral persistence: infections and autoimmune disease. Clin Microbiol Rev.

[17] Cusick MF, Libbey JE, Fujinami RS (2012). Molecular mimicry as a mechanism of autoimmune disease. Clin Rev Allergy Immunol.

[18] Cappello F (2020). Is COVID-19 a proteiform disease inducing also molecular mimicry phenomena?. Cell Stress Chaperones.

[19] Cappello F, Marino Gammazza A, Dieli F, Conway de Macario E, Macario AJL (2020). Does SARS-CoV-2 trigger stress-induced autoimmunity by molecular mimicry? A hypothesis. J Clin Med.

[20] Angileri F, Legare S, Marino Gammazza A, Conway de Macario E, JL Macario A, Cappello F (2020). Molecular mimicry may explain multi-organ damage in COVID-19. Autoimmun Rev.

[21] Hwa KY, Lin WM, Hou YI, Yeh TM (2008). Peptide mimicrying between SARS coronavirus spike protein and human proteins reacts with SARS patient serum. J Biomed Biotechnol.

[22] Tim Chew F, Ong SY, Hew CL (2003). Severe acute respiratory syndrome coronavirus and viral mimicry. Lancet.

[23] Lucchese G, Flöel A (2020). Molecular mimicry between SARS-CoV-2 and respiratory pacemaker neurons. Autoimmun Rev.

[24] Toscano G, Palmerini F, Ravaglia S, Ruiz L, Invernizzi P, Cuzzoni MG, Franciotta D, Baldanti F, Daturi R, Postorino P, Cavallini A, Micieli G (2020). Guillain–Barré syndrome associated with SARS-CoV-2. N Engl J Med.

[25] Gutiérrez-Ortiz C, Méndez A, Rodrigo-Rey S (2020). Miller Fisher syndrome and polyneuritis cranialis in COVID-19. Neurology.

[26] Ibáñez S, Martínez O, Valenzuela F, Silva F, Valenzuela O (2020). Hydroxychloroquine and chloroquine in COVID-19: should they be used as standard therapy?. Clin Rheumatol.

[27] James JA, Kim-Howard XR, Bruner BF, Jonsson MK, McClain M, Arbuckle MR, Walker C, Dennis GJ, Merrill JT, Harley JB (2007). Hydroxychloroquine sulfate treatment is associated with later onset of systemic lupus erythematosus. Lupus.

[28] Schrezenmeier E, Dörner T (2020). Mechanisms of action of hydroxychloroquine and chloroquine: implications for rheumatology. Nat Rev Rheumatol.

[29] Marks M, Marks JL (2016). Viral arthritis. Clin Med (Lond).

